# The Effects of Corticosteroid Injection in the Healthy and Damaged Achilles Tendon Model: Histopathological and Biomechanical Experimental Study in Rats

**DOI:** 10.5146/tjpath.2019.01468

**Published:** 2020-01-15

**Authors:** İlyas Arslan, Istemi Yücel, Turhan Beyza Öztürk, Nazım Karahan, M. Müfit Orak, Ahmet Midi

**Affiliations:** Department of Orthopedics and Traumatology, Fatih Sultan Mehmet Training and Research Hospital, İstanbul, Turkey; Department of 2nd Grade Student, İstanbul Bahçeşehir University Faculty of Medicine, İstanbul, Turkey; Department of Orthopedics and Traumatology, İstanbul Bahçeşehir University Faculty of Medicine, İstanbul, Turkey; Department of Pathology, İstanbul Bahçeşehir University Faculty of Medicine, İstanbul, Turkey

**Keywords:** Corticosteroid, Achilles tendon, Rats

## Abstract

***Objective:*** To show the effects of corticosteroids on inflammatory reactions in the injured Achilles tendon in rats.

***Material and Method:*** Thirty-two adult Wistar Albino rats were used in the study. The rats were divided into 4 groups. In the first group (Intact Saline), saline solution was injected to the intact Achilles tendon. In the second group (Intact Corticosteroid), corticosteroid was injected to the intact tendon. In the third group (Injured Saline), saline solution was injected to the injured Achilles tendon. In the fourth group (Injured Corticosteroid), corticosteroid was injected to the injured tendon. All groups were sacrificed on day 30 and Achilles tendons were taken and prepared for histological and biomechanical evaluation.

***Results:*** According to the biomechanical test; mean load-to-failure of the Intact Saline group was significantly lower than the Intact Corticosteroid (p=0.016), Injured Saline (p=0.001) and Injured Corticosteroid) (p=0.012) groups. According to the histopathological evaluation, tenocyte mean of the Intact Saline group was statistically lower than the Injured Saline and Injured Corticosteroid groups. Tenocyte mean of the Intact Corticosteroid group was statistically significantly lower than the Injured Saline and Injured Corticosteroid groups. The ground substance mean of the Intact Saline group was significantly lower than the Injured Saline and Injured Corticosteroid groups. The ground substance mean of the Intact Corticosteroid group was significantly lower than the Injured Saline and Injured Corticosteroid groups. There was no statistically significant difference between the groups in terms of calcification.

***Conclusion:*** It has been found that there is biomechanical and histopathological significant benefit of intra-tendon corticosteroid administration in the experimentally generated Achilles tendon injury model.

## INTRODUCTION

Achilles tendon is one of the largest, strongest tendons in the body and is the most common tendon that shows pathological situations. Tendinopathies are confronted within a wide range of clinical spectrum, and clinical differentiation between lesions can be challenging for the physician. It is likely that the tendons have pathological changes before rupture and these changes also affect the subsequent healing period. Individuals are often unaware of these pathological changes that do not lead to symptoms and develop achillodynia or tendon ruptures with minimal trauma.

Because the Achilles tendon faces higher *in vivo* stresses than the other tendons, it is the most commonly traumatized and most often ruptured tendon in the human body (1,2). Achilles tendon ruptures are very common especially in sports activities where sudden load is applied to the tendon and then abruptly abolish (2–4).

Tendon ruptures usually occur between the ages of 31-49 and are more frequent in males (1,5). The etiology is not fully understood, but the most common cause is degenerative tendinopathy (1,2,6–8).

There are studies showing that steroid use or direct injection into the tendon region increases the risk (9). In an immobilized tendon, there is a microscopical decrease in cellularity, collagen fibril diameter, collagen cross-links and the entire collagen organization. At the same time, the proteoglycan and water content may also change. Factors affecting tendon healing include age, sex, hormonal status, systemic disease presence, chronic drug use (especially corticosteroid), size of the injured area, crushing injuries that disturb the blood supply of tendon and surrounding tissues, and the applied treatment methods.

The use of corticosteroids in Achilles tendinopathy is still controversial (6–8,10–12). Corticosteroids affect the healing in a negative way by suppressing the inflammatory response, tenocyte proliferation and collagen synthesis. They can cause spontaneous rupture by reducing the tensile strength of the healing tendon (13). According to some studies, corticosteroids delay tendon healing, cause degeneration and impair biomechanical properties (14–21). However, there are also publications reporting no side effects on the tendon and no increase the rate of Achilles tendon rupture (22). There is no clear consensus on the benefits and damages.

Our aim was to show the effect of the anti-inflammatory activity of corticosteroids on the healing of Achilles tendon damage by creating a damaged Achilles tendon model and comparing the biomechanical and histological changes that occur with the changes in the intact Achilles tendon.

## MATERIAL and METHODS

Our study was carried out with approval from the Loc-al Ethics Board (approval no: 2016-16). In the study, 5-7-month-old rats weighing 300-350 grams were used. Thirty-two Achilles tendons of 32 Wistar white female rats with normal activity were included in the study. The rats were housed with 3-4 rats in each cage during the experiment, fed with standard laboratory nutrients. There was no liquid and nutrient restrictions and the rats were divided into 4 groups with each group including 8 animals. The right Achilles tendon was used as the experimental group, while one left Achilles tendon was used as the control group in each group. In the first group (Intact Saline), saline solution was injected to the intact Achilles tendon. In the second group (Intact Corticosteroid), corticosteroid was injected to the intact tendon. In the third group (Injured Saline), saline solution was injected to the injured Achilles tendon. In the fourth group (Injured Corticosteroid), corticosteroid was injected to the injured tendon. Trametasone sodium phosphate (Diprospan, Eczacıbaşı, Turkey) was used for corticosteroid injection. The dose was determined as 0.1 ml (0.7 mg) (23). Rats were maintained in standard laboratory conditions (12 hours day time - 12 hours night time lighting, 20-22 °C room temperature, 50-60% humidity) for a week before starting work, and they were provided with water and food as needed.

### Preparation of Animals

Rats were anesthetized with an injection of 10 mg/kg Rompun (Xylazine, Bayer, Germany) and 100 mg/kg Ketamine HCl (Ketalar, Pfizer, USA) before surgery. Each rat’s right leg was cleaned from hair with a razor blade. The right hind legs were sterilized by providing antisepsis with a solution of 10% Povidone Iodine (Batticon, Adeka Pharmaceutical, Turkey). 10 mg/kg Cefazolin Na (Cefazol, Mustafa Nevzat, Turkey) was intramuscularly injected as a preoperative antibiotic prophylaxis.

### Surgical Technique

**Figure 1:**
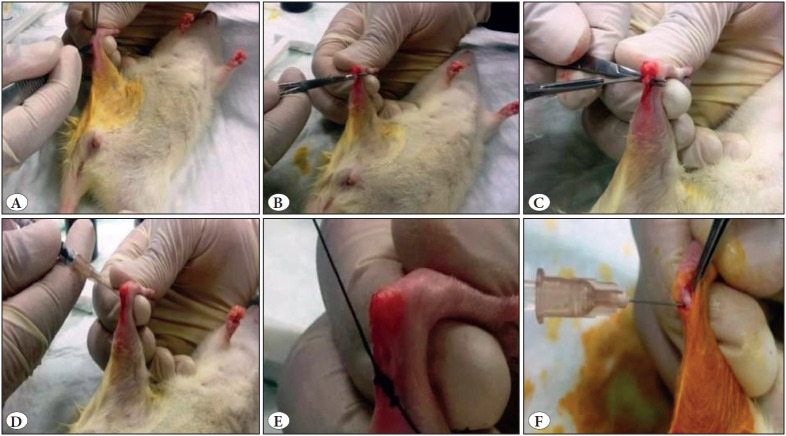
**A)** Skin incision. **B)** Exposure of tendon. **C)** Tendon damage. **D)** Tendon injection (damaged tendon). **E)** Skin closure. **F)** Tendon injection (intact tendon).

A separate surgical instrument was prepared for each experimental animal in a sterile drape, and the rats’ Achilles tendon was cut longitudinally on the skin starting from the calcaneal insertion site and 5 mm lateral to the tendon. (Figure 1). The paratnon and tendon were exposed (Figure 1). The tendon damage model of these animals was based on the Achilles tendon damage model proposed by Akamatsu et al. (23).

According to this article, the tendon was discarded, and the lateral incision made perpendicular to the fibers with a 15 mm lancet at 2.5 mm from the Achilles’ insertion (Figure 1). 0.1 mL (0.7 mg) of betamethasone sodium phosphate (Diprospan, Eczacıbaşı, Turkey) was intrathecal injected to the site where the tendon rupture was made (Figure 1). The skin incision was closed with silk suture No. 4, and it was not dressed (Figure 1). For groups without Achilles tendon damage model, the injection was performed by exposing tendons with a smaller incision (Figure 1).

### Evaluation

**Figure 2:**
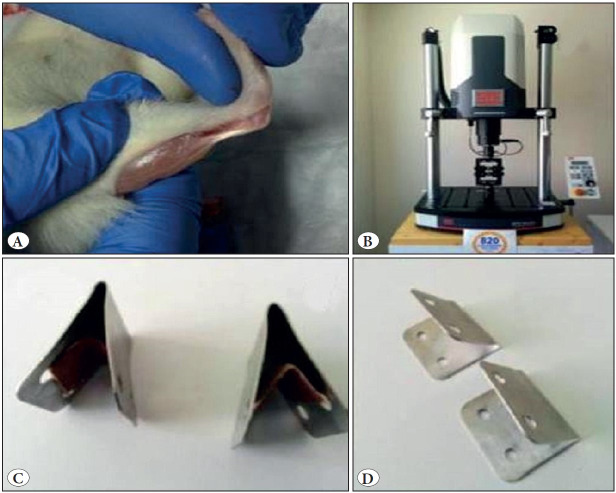
**A)** Exposure of tendon, calcaneus and gastrocnemius. **B)** MTS 858 Mini Bionix II. **C)** Tightening aluminum plates. **D)** Anti-slip sanding-mounted plates.

At the end of the process, rats were released into cages and free access to food and water was provided at room temperature. At the end of 30 days, the experiment was terminated by the sacrificing of the subjects. The right Achilles tendon was subcutaneously skinned 2 cm proximal to the insides, and the tendon was removed by opening the paratenon (Figure 2). To ensure that the Achilles tendon was completely removed, the entire Achilles tendon was excised with a piece of muscle from the triceps surae with a 3 mm bone piece cut from the calcaneus.

### Biomechanical Evaluation

Until biomechanical testing, samples were stored at -20 °C. Biomechanical tests were carried out at Istanbul Technical University Laboratory. Room temperature and humidity were set to 20 °C ± 1 and 40% respectively. Fresh frozen samples were dissolved at room temperature and saline was used to keep them moist. For biomechanical testing, MTS 858 Mini Bionix II (MTS Systems Corporation, USA, 2014) was used (Figure 2).

Aluminum plate specially designed to hold the calcaneus, gastrocnemius and tendon-containing tissues (Figure 2) and abrasive paper (Figure 2) were used.

The system was set to 250 N with a displacement rate of 5 mm / min. Force was applied to the tendon until it splits.

The biomechanical evaluation was performed according to the parameters of maximum load, maximum point elongation and stiffness for each group. The average of each group was taken and recorded.

### Histopathological Evaluation

The right Achilles tendon taken from each subject was kept in neutral formaldehyde solution and sent to the Pathology Laboratory. After 48 hours, the samples were placed into decalcified solution for 15 days. After these procedures, the tendons were cut longitudinally and embedded in paraffin. After this process, five micrometer thick sections were prepared and stained by hematoxylin-eosin stain, and the healing state of the tendon was assessed by light microscopy. Histopathological state was evaluated by x100 magnification. Tenocytes, ground substance, amount of collagen, vascularity (24) and presence of calcification were the parameters assessed; scoring was performed using the semiquantitative scoring system developed by Backman et al. (25).

### Statistical Evaluation

Findings obtained in the study were evaluated using the SPSS Statistics 22 (IBM SPSS, USA) program for statistical analysis. The normal distribution of the parameters was assessed by the Shapiro-Wilk test when the study data were evaluated. The Kruskal-Wallis test was used for comparison of the groups with no normal distribution and the Mann-Whitney U test was used for the determination of the group causing the difference in the comparison of the quantitative data. The Chi-square test was used for comparison of qualitative data. Significance was assessed at **p<0.05.

## RESULTS

### Biomechanical Findings

There was a statistically significant difference between the groups in terms of maximum load averages (*p = 0.002*). As a result of the binary comparisons for differentiation, the maximum load averages in the Intact Saline group were statistically significantly lower than the mean values of Intact Corticosteroid (*p = 0.016*), Injured Saline (*p = 0.001*) and Injured Corticosteroid (*p = 0.012*) groups. There was no statistically significant difference between the other groups in terms of maximum load averages. Statistical evaluation results of biomechanical tests among the groups are shown in Table 1. There was no statistically significant difference between the groups in terms of maximum extension averages. There was no statistically significant difference between groups in terms of stiffness averages.

**Table 1:** Statistical evaluation of maximum load, maximum point elongation and rigidity parameters between groups.

**Groups**	**Maximum load (N)**	**Maximum point elongation (mm)**	**Rigidity (N/mm)**
	**Mean±SD (median)**		
Intact Saline (Group 1)	28.58±8.03 (26.4)	0.98±0.62 (0.7)	45.9±10.71 (45.3)
Intact Corticosteroid (Group 2)	47.22±13.67 (49.4)	1.91±0.85 (1.8)	42.19±13.6 (45.9)
Injured Saline (Group 3)	59.02±9.47 (57.4)	1.63±0.47 (1.5)	44.88±15.4 (44.1)
Injured Corticosteroid (Group 4)	50.55±17.46 (46.9)	1.58±0.9 (1.4)	49.57±16.22 (44.6)
p	0.002*	0.064	0.949

Kruskal-Wallis test *p<0.05.

### Histological Findings

**Figure 3:**
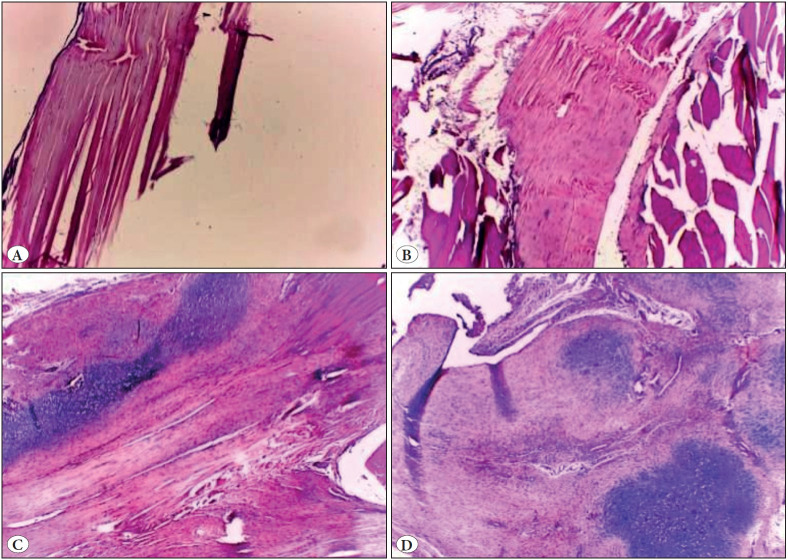
**A)** Normal appearance of the tendon in the intact saline group and **B)** cortisone group (H&E; x40). **C)** Chondroid differentiation in the damaged cortisone group (H&E; x40). **D)** Vascularity in the injured saline group (H&E; x40).

**Figure 4:**
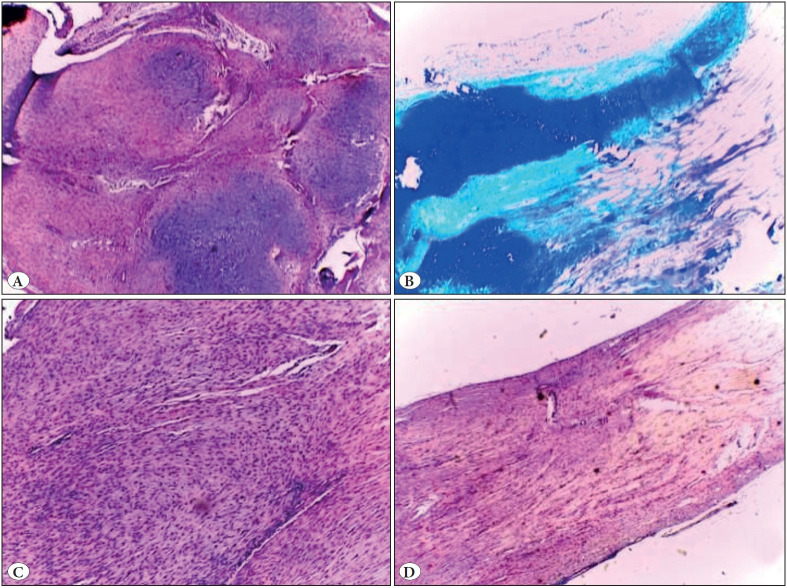
**A)** Chondroid differentiation (H&E; x40). **B)** Mucinous matrix (Alcian blue; x40). **C)** Enlargement and rounding of cell nuclei in the damaged saline group (H&E; x100). **D)** Close to normal appearance in the damaged cortisone group (H&E; x40).

All rat right legs underwent corticosteroid injection and their controlled left legs were evaluated histopathologically (Table 2). (Figure 3 , Figure 4).

**Table 2:** Statistical evaluation of histological parameters between groups.

**Histopathologic** **Parameters**	**Intact Saline (Group 1)**	**Intact Corticosteroid (Group 2)**	**Injured Saline (Group 3)**	**Injured Corticosteroid (Group 4)**	**P**
Mean±SD (median)					
Tenocyte	0±0 (0)	0.13±0.35 (0)	2±0.93 (2)	1.88±0.83 (2)	^1^0.001*
Ground substance	0±0 (0)	0±0 (0	1.88±1.13 (2)	2±1.07 (2)	^1^0.001*
Collagen	0±0 (0)	0±0 (0	1.63±0.92 (2)	1.5±1.07 (1)	^1^0.001*
Vascularity	0±0 (0)	0±0 (0	1.5±1.2 (1.5)	1.25±1.28 (1)	^1^0.001*
Calcification n (%)					
Negative	4 (50%)	6 (75%)	6 (75%)	7 (87.5%)	^2^0.401
Positive	4 (50%)	2 (25%)	2 (25%)	1 (12.5%)	

^1^Kruskal-Wallis test, ^2^Chi square test, *p<0.05.

There was a statistically significant difference between groups in terms of tenocyte mean values (p = 0.001). As a result of the binary comparisons for discrimination, tenocyte averages in the Intact Saline group were statistically significantly lower than the Injured Saline and Injured Corticosteroid groups (*p = 0.001*, *p<0.05, respectively). Tenocyte averages in the Intact Corticosteroid group were statistically significantly lower than those in the Injured Saline and Injured Corticosteroid groups (p = 0.001; *p<0.05, respectively).

There was no statistically significant difference in tenocyte averages between the other groups. There was a statistically significant difference between the groups in terms of the average of the ground substance (*p = 0.001*). As a result of the binary comparisons made to determine the difference; The mean values of the ground substance in the Intact Saline group were statistically significantly lower than the mean values of the Injured Saline and Injured Corticosteroid groups (p = 0.001, *p<0.05, respectively). The mean values of the ground substance in the Intact Corticosteroid group were statistically significantly lower than those of the Injured Saline and Injured Corticosteroid groups (p = 0.001, *p<0.05, respectively). There was no statistically significant difference in the mean of the ground substance among the other groups. There was a statistically significant difference in collagen averages between the groups (*p = 0.001*). As a result of the binary comparisons made to determine the difference; the collagen averages in the Intact Saline group were statistically significantly lower than the Injured Saline and Injured Corticosteroid groups (p = 0.001, *p<0.05, respectively).

The collagen averages in the Intact Corticosteroid group were statistically significantly lower than those of the Injured Saline and Injured Corticosteroid groups (p = 0.001, *p<0.05, respectively). There was no statistically significant difference in collagen averages between the other groups.

There was a statistically significant difference between the groups in terms of vascularity averages (*p = 0.001*). As a result of the binary comparisons made to determine the difference;

The mean vascularity in Intact Saline group was found to be statistically significantly lower than the mean values of the Injured Saline (*p = 0.004*) and Injured Corticosteroid (*p = 0.010*) groups (*p<0.05). The vascularity averages in the Intact Corticosteroid group were statistically significantly lower than the mean values of Injured Saline (*p = 0.004*) and Injured Corticosteroid (*p = 0.010*) groups (*p<0.05). There was no statistically significant difference in vascularity averages between the other groups. There was no statistically significant difference in calcification distribution ratios between the groups.

## DISCUSSION

In our study, we aimed to observe the positive or negative effects of corticosteroids on healing with the reducing effect of inflammatory actions in the damaged tissue and to show whether corticosteroids could be used in injured Achilles tendon in humans. According to the biomechanical test, the maximum load averages in the Intact saline group were statistically significantly lower than the mean values of the Intact Corticosteroid, Injured Saline and Injured Corticosteroid groups. In histopathological examination, tenocyte averages in the first group were statistically significantly lower than the mean of the Injured Saline and Injured Corticosteroid groups. The mean values of the ground substance in the Intact saline group were statistically significantly lower than those of the Injured Saline and Injured Corticosteroid groups.

In our study, according to the biomechanical test and histopathological examination, corticosteroids had positive effects on healing in the injured Achilles tendon.

Treatment of Achilles tendinosis is still a subject of debate and research is underway. In animal studies, there are more than one way to create tendon degeneration (1). We believe that the most appropriate one among these methods is the Achilles tendon damage model proposed by Akamatsu et al. (23). We preferred mechanical tendon damage instead of chemical damage as substances that chemically damage the Achilles tendon would interact with our corticosteroids. The partial/complete rupture and repair model has been used in many studies. However, in this model, it was concluded that the repair of the lesion could not mimic the incidence of Achilles tendinosis and subsequent healing after rupture due to degeneration. Another controversial issue is whether corticosteroids will be administered intratendinously or into the paratenon. In relation to this, there are many studies with various applications. In many experimental studies, it has been found that the intratendinous administration of steroid leads to a reduction in tendon strength by producing significant amounts of collagen necrosis (19). Experimental studies have shown that corticosteroids cause rupture risk (20) and degeneration even weeks later, leading to hypoxic degenerative changes, especially when administered intratendinously. Applications into the paratenon are controversial (20). It has been reported that corticosteroids may cause spontaneous tendon rupture with destruction of the wound while reducing the symptoms present in the tendon with an anti-inflammatory effect (21). Several animal studies on the delaying effects of local corticosteroid injection on tendon healing have been conducted. 0.1 ml (0.7 mg) of corticosteroid injection is equivalent to a normal 70 kg human dose (24). Corticosteroid injection has been proven to be applicable to healthy tendons to model experimental tendon degeneration. 0.1 mg/kg normal human dose was also used in animal studies (24).

In the light of this literature information, we found it appropriate to use the same dose. In this part of the study, the tendon was disrupted before the injection into the intact Achilles tendon in order to make sure that the injection was intratendinous and corticosteroid injected. In Achilles tendinosis healing, it is important that the functional performance of the individual and success/failure decision in treatment as well as the biomechanical properties of the Achilles tendinosis is based on this clinical measure. In clinical work, Achilles tendon function can be measured with the aid of static weight and dynamometer. Additional biomechanical studies have shown that the functional recovery and mechanical recovery of the recovering tendon are similar (22).

In the biomechanical evaluation of the tendons obtained after sacrification of the rats, the maximum load averages in the Intact Saline group were statistically significantly lower than in the other groups (26).

The gross macroscopic appearance of the Intact Saline was already thin and consistently soft compared to other groups. This group concluded that it affected the strength of damaged tendons and lack of fibrosis in the intact tendon group treated with corticosteroids (27).

In order to prevent this, it is reported that shortening of the 1-month waiting period after the experiment can lead to more accurate results in the case of shorter recovery time (28). Although the etiology of Achilles tendinosis is still being discussed, many studies have been conducted to elucidate the inflammatory and degenerative properties of the pathology. Although the underlying pathology of chronic tendon lesions is degenerative, inflammation may also be present in acute pathologies (23).

When this study was planned, semiquantitative histopathologic examination taking into account the tendon degeneration after tendon injury application and the inflammatory changes was planned. In this study, a method including parameters of tenocyte, ground substance, amount of collagen, vascularity and calcification suggested by Aydın et al. (24), which shows intratendinous degeneration in histopathologically evaluated tendon sections, was used.

Findings in this study are consistent with the literature. The mean of the ground substance (extracellular matrix elements secreted by the fibroblasts in connective tissue) in the groups of intact tendon injections was statistically significantly lower than that of the damaged tendon groups. This result is related to the increase in the amount of protein in repair tissue (24). Similarly, the mean amount of collagen in groups of intact tendon injections was found to be statistically significantly lower than that of damaged tendon groups.

In one study, thrombocyte-rich fibrin was shown in supraspinatus tendons in rats and helped heal tendon-bone. In this study, test groups with and without fibrin were compared. It was shown that those using fibrin are stronger. Histological studies have also shown that healing response and fibrous tissue production are greater in the fibrin group. It has been suggested that this is due to collagen production in repair tissue (25). Also, the vascularity averages in groups of intact tendon injections were found to be statistically significantly lower than those of the damaged tendon groups. However, Gigliotti et al. have shown the exact opposite in their work (29). They took a biopsy from torn and firm rotator cuff tendons in the shoulder during arthroscopy and histologically examined them. Tendon injury model used in our study is more acute period compared in this study. The chronic process may be considered to reduce vascularity, but this is a different research topic. Although O’Brien et al. (30) showed that heterotropic ossification and calcification were triggered and increased by trauma in the tendon-bone attachment regions, no statistically significant difference was found between the groups in terms of calcification in our study. On the other hand, there was a statistically significant difference in terms of tenocyte, ground substance, amount of collagen and vascularity in our study. This is because O’Brien and his colleagues carried out studies on the bone-tendon junction rather than the intratendon area.

## CONCLUSION

The use of corticosteroids is still controversial although it is one of the most preferred drugs in the treatment of Achilles tendinopathy. There is still no consensus on the form of application, dose and total treatment duration for tendons. There are many studies on tendinopathy treatment. In a literature review, we found that these studies were performed on intact tendons. Therefore, we decided to choose a damaged tendon. At the end of our study, we obtained biomechanical and histopathologic data showing that the maximum load averages of damaged tendon groups were higher. We think that this tendency is directly proportional to the fibrosis of the healing tendon.

We believe that we should wait for a shorter period as the one-month waiting period prior to sacrificing the rats is long and results in excessive fibrosis. We also grossly observed this. The damaged tendons were macroscopically thicker and harder and thus more difficult to break.

The fact that the changes were not shown at the molecular level and the results of the studies in the literature differed from ours are considered as limitations of the present study.

The strengths of the study were that biomechanical and histopathological evaluations were performed together and histopathological evaluation was performed by a pathologist.

## Conflict of Interest

The authors declare no conflict of interest.
